# Effects of a support group leader education program jointly developed by health professionals and patients on peer leader self-efficacy among leaders of scleroderma support groups: a two-arm parallel partially nested randomised controlled trial

**DOI:** 10.1186/s13023-022-02552-x

**Published:** 2022-10-28

**Authors:** Brett D. Thombs, Brooke Levis, Marie-Eve Carrier, Laura Dyas, Julia Nordlund, Lydia Tao, Kylene Aguila, Angelica Bourgeault, Violet Konrad, Maureen Sauvé, Kerri Connolly, Richard S. Henry, Nora Østbø, Alexander W. Levis, Linda Kwakkenbos, Vanessa L. Malcarne, Ghassan El-Baalbaki, Marie Hudson, Amanda Wurz, S. Nicole Culos-Reed, Robert W. Platt, Andrea Benedetti, SPIN-SSLED Support Group Leader Advisory Team, Stephen Elrod, Catherine Fortuné, Amy Gietzen, Karen Gottesman, Karen Nielsen, Michelle Richard, Ken Rozee, Nancy Stephens

**Affiliations:** 1 Lady Davis Institute for Medical Research, Jewish General Hospital, 3755 Cote Ste Catherine Road, Montreal, QC H3T 1E2 Canada; 2 Department of Psychiatry, McGill University, Montreal, QC Canada; 3 Department of Epidemiology, Biostatistics, and Occupational Health, McGill University, Montreal, QC Canada; 4 Department of Medicine, McGill University, Montreal, QC Canada; 5 Department of Psychology, McGill University, Montreal, QC Canada; 6 Biomedical Ethics Unit, McGill University, Montreal, QC Canada; 7 Centre for Prognosis Research, School of Medicine, Keele University, Staffordshire, UK; 8 National Scleroderma Foundation, Michigan Chapter, Southfield, MI USA; 9 Sclérodermie Québec, Sherbrooke, QC Canada; 10 Scleroderma Society of Ontario and Scleroderma Canada, Hamilton, ON Canada; 11 National Scleroderma Foundation, Danvers, MA USA; 12 Department of Biostatistics, Harvard T.H. Chan School of Public Health, Boston, MA USA; 13 Department of Clinical Psychology, Radboud University, Nijmegen, The Netherlands; 14 Department of Medical Psychology, Radboud Institute for Health Sciences, Radboud University Medical Center, Nijmegen, The Netherlands; 15 Department of IQ Healthcare, Radboud Institute for Health Sciences, Radboud University Medical Center, Nijmegen, The Netherlands; 16 Department of Psychiatry, Radboud Institute for Health Sciences, Radboud University Medical Center, Nijmegen, The Netherlands; 17 Department of Psychology, San Diego State University, San Diego, CA USA; 18 San Diego Joint Doctoral Program in Clinical Psychology, San Diego State University/University of California, San Diego, CA USA; 19 Department of Psychology, Université du Québec à Montréal, Montreal, QC Canada; 20 School of Kinesiology, University of the Fraser Valley, Abbotsford, BC Canada; 21 Faculty of Kinesiology, University of Calgary, Calgary, AB Canada; 22 Department of Oncology, Cumming School of Medicine, Calgary, AB Canada; 23 Department of Psychosocial Resources, Tom Baker Cancer Centre, Alberta Health Services, Calgary, AB Canada; 24 Respiratory Epidemiology and Clinical Research Unit, McGill University Health Centre, Montreal, QC Canada; 25 National Scleroderma Foundation of Southern California Patient Support Group, Bakersfield, CA USA; 26 Ottawa Scleroderma Support Group, Ottawa, ON Canada; 27 Scleroderma Foundation, Danvers, MA USA; 28 National Scleroderma Foundation, Los Angeles, CA USA; 29 Scleroderma Society of Ontario, Hamilton, ON Canada; 30 Scleroderma Atlantic, Halifax, NS Canada; 31 Michigan Patient Group, Detroit, MI USA

**Keywords:** Chronic diseases, Peer support, Support groups, Systemic sclerosis, Rare diseases

## Abstract

**Background:**

More people with rare diseases likely receive disease education and emotional and practical support from peer-led support groups than any other way. Most rare-disease support groups are delivered outside of the health care system by untrained leaders. Potential benefits may not be achieved and harms, such as dissemination of inaccurate information, may occur. Our primary objective was to evaluate the effects of a rare-disease support group leader education program, which was developed collaboratively by researchers, peer support group leaders, and patient organization leaders, compared to waitlist control, on peer leader self-efficacy among scleroderma support group leaders.

**Methods:**

The trial was a pragmatic, two-arm partially nested randomised controlled trial with 1:1 allocation into intervention or waitlist control. Eligible participants were existing or candidate peer support group leaders affiliated with a scleroderma patient organization. Leader training was delivered in groups of 5–6 participants weekly for 13 weeks in 60–90 min sessions via the GoToMeeting® videoconferencing platform. The program included 12 general leader training modules and one module specific to scleroderma. Primary outcome was leader self-efficacy, measured by the Support Group Leader Self-efficacy Scale (SGLSS) immediately post-intervention. Secondary outcomes were leader self-efficacy 3 months post-intervention; emotional distress, leader burnout, and volunteer satisfaction post-intervention and 3 months post-intervention; and program satisfaction among intervention participants post-intervention.

**Results:**

One hundred forty-eight participants were randomised to intervention (N = 74) or waitlist (N = 74). Primary outcome data were provided by 146 (99%) participants. Mean number of sessions attended was 11.4 (standard deviation = 2.6). Mean program satisfaction score (CSQ-8) was 30.3 (standard deviation = 3.0; possible range 8–32). Compared to waitlist control, leader self-efficacy was higher post-intervention [SGLSS; 16.7 points, 95% CI 11.0–22.3; standardized mean difference (SMD) 0.84] and 3 months later (15.6 points, 95% CI 10.2–21.0; SMD 0.73); leader volunteer satisfaction was significantly higher at both assessments, emotional distress was lower post-intervention but not 3 months later, and leader burnout was not significantly different at either assessment.

**Conclusions:**

Peer support group leader education improved leader self-efficacy substantially. The program could be easily adapted for support group leaders in other rare diseases.

*Trial registration:* NCT03965780; registered on May 29, 2019.

**Supplementary Information:**

The online version contains supplementary material available at 10.1186/s13023-022-02552-x.

## Background

People with rare diseases face the same challenges as those with more common diseases plus other unique challenges, including limited access to disease education and support [1–5]. Professionally organized support services for people with common diseases are often available through the healthcare system [6, 7], but they are not typically available in rare diseases [8]. As a result, many people with rare diseases look to peer-led support groups for education, to learn skills to better manage their condition, and as a source of social support, [9–13] which may include emotional support and companionship, as well as informational and tangible task-related support [14–16]. Social support is robustly associated with quality of life in the general population and among people with medical conditions [14–17], including rare diseases, particularly emotional and companisonship support [15, 18, 19]. More than half of people with rare diseases, however, report that their social support needs are not adequately met [20, 21]. Peer support groups have been suggested as a way to address this gap, but many people with rare diseases are not informed about or cannot access support groups [21–24].

Peer support groups may improve patient experiences, healthy behaviours, and psychological outcomes among people with rare diseases and other chronic conditions [25]. A 2003 population-based survey from the United States reported that 24% of over 1800 cancer survivors and 15% of almost 5000 people with noncancerous chronic conditions had participated in support groups [26]. Support group participation is likely more common today due to a greater emphasis on patient empowerment and peer support and increased accessibility via virtual options [27–31], and it may be even more common in rare diseases than in more common conditions [9–13]. Many rare-disease peer support groups are sponsored by local, regional, or national patient organizations, and most are organized and delivered outside of the health care system and without collaboration from trained health care professionals [9–13, 31–34]. Many health professionals, however, are reluctant to recommend support groups to patients and some people with rare diseases do not attend due to concerns that the groups may be poorly organized or overly negative and that some disseminate inaccurate information about the health condition of members, its management, and health care professionals who provide care [22–24, 33–39].

Systemic sclerosis (SSc) is a rare, chronic, autoimmune disease characterized by abnormal fibrotic processes that affect multiple organ systems, including the skin, lungs, gastrointestinal tract, and heart [40]. SSc patient organizations around the world sponsor peer-led support groups (see Additional file 1: S1 for examples), but many initiated support groups are not sustained, and some people do not attend ongoing groups due to concerns about how they are conducted [22–24, 33–39]. SSc peer support group leaders report challenges that include practical difficulties (e.g., lack of resources, poor coordination with health care professionals), difficulties with group leadership tasks (e.g., managing complex group dynamics, dealing with the worsening health or death of group members), and personal challenges (e.g., managing one’s own health condition while supporting others) [12, 32].

Peer leader education and training could improve the quality of education and support provided by support group groups, reduce harms from dissemination of inaccurate information and inappropriate health-related advice, and reduce burden on leaders. A 2018 systematic review [41], however, identified only one trial [42] that compared an informational website, online discussion forum, and two-day educational workshop (N = 29) to the website and discussion forum alone (N = 23) among cancer support group leaders. No differences in leader confidence or self-efficacy were found, but the small sample size, minimal documentation of the training provided, and methodological limitations reduce confidence that findings would apply to other settings. We did not identify any more recently conducted trials.

The Scleroderma Patient-centered Intervention Network (SPIN) partnered with SSc patient organization leaders and an 11-person Support Group Leader Advisory Team to develop the Scleroderma Support group Leader EDucation (SPIN-SSLED) Program, a 3-months-long weekly group videoconference training program designed to improve leadership skills and self-efficacy, reduce burnout, and reduce emotional distress among support group leaders. The program includes 12 general peer-support group leader education modules and one module on SSc knowledge and education. After initial development, the program was tested in a feasibility trial [43].

Self-efficacy refers to one’s belief in their ability to meet challenges and perform behaviours necessary to achieve desired outcomes [44]. Among people with chronic diseases, self-efficacy for carrying out health behaviours is associated with improved disease management and better outcomes [45]. Communication skills training that improves health care provider self-efficacy is associated with better patient care ratings [46], and, in education, teacher self-efficacy is associated with better student outcomes [47]. Support group leader self-efficacy reflects a leader’s confidence that they have the knowledge and skills necessary to perform important leadership tasks, which would similarly be expected to lead to more effective education and support for support group members [48].

The primary objective was to evaluate the hypothesis that the SPIN-SSLED Program would improve support group leadership self-efficacy, measured by the Support Group Leader Self-efficacy Scale (SGLSS) [48], immediately post-intervention compared to waitlist control. Secondary objectives were to evaluate the program’s effect on peer leadership self-efficacy 3 months post-intervention and on emotional distress, measured by the Patient Health Questionnaire-8 (PHQ-8) [49]; burnout, measured by the Oldenburg Burnout Inventory (OLBI) [50]; and leader volunteer satisfaction, measured by the Participation Efficacy subscale of the Volunteer Satisfaction Index (VSI) [51] immediately post-intervention and 3 months later. We also evaluated participant satisfaction for intervention participants via the Client Satisfaction Questionnaire-8 (CSQ-8) [52] immediately post-intervention.

## Methods

The SPIN-SSLED Trial was a pragmatic, two-arm parallel partially nested randomised controlled trial (PN-RCT) [53]. Pragmatic RCTs are intended to replicate real-world conditions and support a decision on whether an intervention should be provided [54–57]. Randomisation was 1:1 to intervention or waitlist control. A PN-RCT design [53] was used because intervention participants were clustered in groups, whereas waitlist participants were not grouped. We used a waitlist control because patient organization partners were invested in providing program access. Ethics approval was obtained from the Research Ethics Committee of the Centre intégré universitaire de santé et de services sociaux du Centre-Ouest-de-l'Île-de-Montréal (#2020-1780). The trial was registered prior to enrolling participants (NCT03965780), and a protocol was submitted for publication prior to enrolling participants [58]. It is reported in accordance with standards articulated in the Consolidated Standard of Reporting Trials (CONSORT) statement [59] and CONSORT extensions for nonpharmacologic trials [60], cluster trials [61], pragmatic trials [54], and e-health trials [62].

### Trial eligibility

Eligible trial participants had to be existing support group leaders affiliated with a SSc patient organization or candidate support group leaders who an organization confirmed would start a support group following training. Participants had to confirm their ability to use the Internet to participate in training sessions and to complete study measures online in English or French. To avoid contamination, for existing support groups with more than one leader, only one leader could enroll.

### Recruitment and enrollment

Patient organization partners, including Scleroderma Canada and Canadian provincial organizations, the National Scleroderma Foundation in the United States, Scleroderma & Raynaud’s UK, Scleroderma Australia and Australian state organizations, and Scleroderma New Zealand contacted current and candidate leaders to describe the SPIN-SSLED Program and then provided the SPIN team with a list of interested leaders. In addition, we advertised the trial through SPIN’s Facebook and Twitter accounts and on SPIN’s website so that potential candidate leaders not known to their organizations could contact their patient organizations about their interest and to reach current or candidate leaders from other patient organizations. We also created a webpage with a brief introductory video on the program, video and written testimonials from support group leaders who participated in the SPIN-SSLED Feasibility Trial, and information about the program structure and content [63].

SPIN-SSLED personnel emailed potentially interested participants a Qualtrics survey link containing more information about the trial, including that participants would be randomly assigned to the intervention or to a waitlist control, and the consent form. Potential participants could consent electronically or request to be contacted. Consented participants provided information about their support group experience (e.g., years of experience or candidate leader), language preference (English or French), and days and times when they could attend training sessions. For existing support groups with co-leaders, the co-leaders were asked to specify primary or secondary status for the trial, and secondary leaders were only enrolled if the primary leader’s day and time availabilities did not match any training groups provided in the trial.

### Randomisation and masking

Training groups were conducted in 3 months waves across the trial period with the number of groups in each wave determined by the number of available participants. At the beginning of each wave, enrolled participants were entered into pools based on language and scheduling availability. De-identified codes for participants in each pool were provided to an external centralized randomisation service, the Griffith Randomisation Service (Queensland, Australia; https://randomisation.griffith.edu.au/), to ensure allocation sequence concealment [64]. Starting with the largest pool, the service randomly selected the largest possible even number of participants (10 or 12 participants) then randomly allocated half (5–6 participants) to intervention and half (5–6 participants) to control via block randomisation. Blocks were stratified by current and candidate leaders with no more than 4 candidate leaders randomised from any pool (2 per intervention group). After randomisation, participants were sent their intervention or waitlist assignment by email; this occurred 2 weeks prior to initiating intervention sessions. Within a week of the assignment emails, those allocated to intervention received a second email with their schedule, the program manual, and information on how to login to the videoconferencing system and online chatroom.

Participants and research staff were not blinded to intervention status. In contrast to explanatory trials that are designed to test intervention effects under ideal circumstances, pragmatic trials are done to provide information on the relative merits of real-world alternatives delivered in routine conditions and focus on maximizing external validity. Indeed, blinding in most pragmatic trials is discouraged so that all effects, including non-specific effects, of providing an intervention can be tested. The effects of receiving the SPIN-SSLED Program compared to not receiving an intervention, in this context, are not limited to its active ingredients but include other components, such as participant expectations and interactions with training personnel, as would occur in the delivery of the program outside of a trial [54–57].

### Intervention and comparator

The SPIN-SSLED Program is a group videoconference-based intervention that was developed based on review of support group leader manuals from other organizations; interviews and surveys with SSc support group leaders, support group participants, and support group non-participants [10–12, 23, 24]; and input from SSc patient organization leaders and an 11-person Support Group Leader Advisory Team. Minor content modifications to the original program were made based on feedback from feasibility trial participants [43]. Training groups were offered in English or French.

Groups of 5–6 participants met weekly for 13 weeks in 60–90 min sessions via the GoToMeeting® videoconferencing platform. Participants received a SPIN-SSLED Program Manual that summarizes didactic material from sessions. Instructors facilitate each session using the SPIN-SSLED Instructor Manual, which includes guidance on introducing material and discussion prompts, along with PowerPoint slides to summarize key material. See Additional file 2: S2 for manuals and in-session materials. All English-language materials were translated into French by a research team member and reviewed by multiple bilingual team members. English-language training groups were facilitated by a master’s level clinical social worker with 28 years of experience and over 10 years training SSc support group leaders. The French-language group was facilitated by a graduate of the SPIN-SSLED Program with an education background and over 5 years of experience leading a SSc support group who was trained and supervised by the English-language group trainer.

The SPIN-SSLED Program uses problem-based learning, which is a learner-centred approach that integrates theory and practice by providing necessary knowledge and skills, presenting complex, real-world problems, and working to identify approaches to solving problems [65, 66]. Each module, or learning session, introduces and provides information on the module topic. In modules that involve managing group or individual interactions, videos previously recorded with members of the Support Group Leader Advisory Team show support group leaders faced with a challenging problem or situation like those that occur in actual support groups. Each module includes guided discussion among participants about the material and possible approaches and solutions to challenging situations. Trainers emphasize the role of group leaders in ensuring that information disseminated is from credible sources and in establishing a culture where experiences of group members are shared but not health or medical advice.

The program includes 12 general leader education modules plus one module designed specifically to provide information on SSc. Module topics include (1) The Leader’s Role; (2) Starting a Support Group; (3) Structuring a Support Group Meeting; (4) Scleroderma 101; (5) Successful Support Group Culture; (6) Managing Support Group Dynamics; (7) Loss and Grief: The Support Group Leader; (8) Loss and Grief: Supporting Group Members; (9) Advertising and Recruitment for the Support Group; (10) Continuity of the Group; (11) Supporting Yourself as a Leader; (12) Remote Support Groups; and (13) Transitions in Support Groups. See Table 1 for more detail.

**Table 1:** SPIN-SSLED program module overview

Module title	Module description
1. The Support Group Leader’s Role	This module discusses the benefits of being a support group leader, the expectations of what the role of leader involves (e.g., facilitation of meetings and interactions but not giving medical advice), and tips for being an effective and supportive leader
2. Starting a Support Group	This module discusses the purpose of a support group, what people with scleroderma hope to gain from support group, why some do not attend, establishing leadership (e.g., one leader, co-leader), membership (e.g. patients only, open to family, and friends), logistics of starting a group (e.g., time, place and meeting duration)
3. Structuring Support Group Meetings	This module discusses formatting group meetings and how to successfully integrate both educational activities with emotional and practical support for members, setting up a meeting agenda
4. Scleroderma 101	This module shows a filmed conference presentation by a physician specialized in scleroderma who explains the different types of scleroderma, symptoms, causes, medical treatments, and alternative approaches. The module also includes tips to evaluate credibility of information sources on the Internet
5. Successful Support Group Culture	This module discusses the importance of establishing expectations and guidelines for the support group with members, the importance of confidentiality, how to create and maintain positive and productive support group culture using (1) encouraging statements, (2) open-ended questions, (3) body language, (4) linking similar experiences between members, and (5) summarizing discussions. This module uses video vignettes to illustrate these techniques
6. Managing Group Dynamics	This module discusses managing difficult support group dynamics such as members who are “quick fixers”, overly talkative, how to maintain a positive group environment, conflict management and resolution for minor and larger issues. The topics discussed also include overly shy members, chronically negative members and members that bring unsubstantiated, potentially misleading medical information to the group. This module uses video vignettes to illustrate these techniques
7. Loss and Grief: The Support Group Leader	This module defines loss, bereavement, grief and mourning. The module discusses the styles of processing loss and grief, healing strategies and how to deal with loss and grief as a support group leader. The importance of creating a loss and grief plan for the support group is also discussed
8. Loss and Grief Scleroderma: Supporting Group Members	The module discusses how grief may be experienced in newly diagnosed members, common cognitive and emotional reactions that people can experience in response to a diagnosis as well as reaching acceptance and adaptation with respect to your diagnosis
9. Advertising and Recruiting for the Support Group	This module discusses how to advertise and promote a support group, how to recruit new members for support groups on ongoing basis, advertising through patient organizations and strategies to retain members
10. The Continuity of the Group	This module discusses the importance of understanding and overcoming reluctance in seeking feedback, the importance of feedback in the support group experience, how to obtain and respond to feedback, how to identify reasons why members may stop attending meetings and strategies to help maintain membership, how to keep members engaged and move your support group forward by making changes
11. Supporting Yourself as a Leader	This module discusses understanding what leader burnout is, understanding why it can happen and what the warning signs are, understanding the best way to address burnout including identifying methods of coping, understanding at what point it may be best for a leader to step down from their role, strategies to prevent experiencing leader burnout
12. Remote Support Groups	This module discusses the benefits of an online support group, finding the right technology, scheduling and programming, advertising and reaching your target audience, tips for successful online meetings
13. Transitions in Support Groups	This module discusses how to handle transitions in support group leadership, discussing leaders’ experiences with the SPIN-SSLED Program and some meaningful takeaways. This module also discusses the transition from the weekly program training sessions to the optional monthly teleconference meetings

In addition to the videoconference training sessions, participants had access to a secure, monitored online forum to interact with other program participants about program content and to a resource centre with video presentations by SSc expert physicians and other educational material for use in their support groups.

Participant attendance at each session was recorded. All training sessions were video-recorded, and a randomly selected sample of 25% were audited for adherence to planned session components. Consistent with best-practice recommendations for evaluating treatment fidelity [67, 68], this was done using a checklist that reflected the specific components of each module. To minimize contamination risk, participants randomised to the intervention were asked not to share program material or discuss sessions with anybody outside of their intervention group.

### Waitlist

Waitlist participants were informed that they would be offered access to the SPIN-SSLED Program following their 3-months post-intervention outcome assessment. During the trial period, they only received reminders to complete trial measures.

### Trial outcomes

On the date of each intervention group’s last session and 3-months later, intervention and paired waitlist participants were sent email invitations to complete trial measures online via Qualtrics. Measures could be completed up to 14 days post-invitation; email reminders were sent 3, 5, 7, 10, and 14 days after initial invitations, and phone calls were also made at days 5, 7, 10, and 14 if measures had not been completed. All measures were available in English and French or translated into French by SPIN researchers using forward–backward translation methods with expert verification and patient testing [69]. Detailed information on outcome measures is available in Additional file 3: S3.

The primary outcome analysis compared SGLSS [48] scores between group leaders in the intervention versus waitlist immediately post-intervention. The SGLSS is a 32-item scale designed to assess support group leader confidence to successfully perform leader tasks (e.g., organizational skills), manage group and interpersonal interactions, and balance group leadership and self-care needs. Items reflect core SPIN-SSLED Program content. The SGLSS utilizes a 6-point Likert scale ranging from 1 (strongly disagree) to 6 (strongly agree), with higher total scores (possible range 32–192) indicating greater self-efficacy. The SGLSS was previously validated in a sample of 102 SSc support group leaders [48].

Secondary outcomes included the SGLSS 3 months post-intervention and other outcome measures immediately post-intervention and 3 months post-intervention, including emotional distress (PHQ-8) [49] among all participants, leader burnout (OLBI) [50] and leader satisfaction (VSI—participation efficacy subscale) [51] among existing support group leaders, and satisfaction with the SPIN-SSLED Program (CSQ-8) [52] among intervention participants.

- *Emotional distress* The 8-item PHQ-8 [49] measures depression symptoms over the last 2 weeks. Higher scores (range 0–24) reflect greater symptom levels.
- *Leader burnout* The 16-item OLBI [50] assesses current exhaustion and disengagement due to burnout. Higher scores (range 16–64) reflect greater burnout.
- *Leader satisfaction (participation efficacy)* The 7 items of the participation efficacy subscale of the VSI [51] measure support group leader satisfaction that they are benefitting others in their role. Higher scores (range 7–49) indicate greater satisfaction.
- *Satisfaction with SPIN-SSLED Program* The 8-item CSQ-8 [52] assesses satisfaction with health services and was adapted for SPIN-SSLED. Total scores range from 8 to 32 with higher scores reflecting greater satisfaction.

### Data analysis

The full statistical analysis plan can be found in Additional file 4: S4.

Meta-analyses of effects of patient and caregiver training programs on knowledge acquisition and self-efficacy in implementing skills have reported standardized mean difference (SMD) effect sizes of 0.58–0.94 [70–74]. For an assumed effect size of SMD = 0.70, two-tailed α = 0.05, and intra-class correlation coefficient (ICC) of 0.05 [75–77], N = 75 participants with outcome data provides ≥ 80% power. Assuming 20% loss to follow-up would require randomising 94 participants. For secondary outcomes (e.g., burnout, emotional distress), SMD = 0.50 is considered a clinically meaningful effect size for similar patient-reported health outcomes, including depressive symptoms [78]. For SMD = 0.50, two-tailed α = 0.05, and ICC of 0.05, N = 146 would provide ≥ 80% power. Assuming 20% loss to follow-up, we sought to randomise 182 support group leaders to have outcome data from 146 participants.

All outcome analyses were conducted in R (R version 3.6.1; R Studio version 1.2.1335). We used an intent-to-treat analysis to estimate score differences between intervention and waitlist participants with a linear mixed-effects model fit using the lmer function in lme4 [79]. Score differences and Hedges’ g SMD effect sizes were presented with 95% confidence intervals (CIs). For all models, with one exception, to account for clustering in the blocked PN-RCT design, we fit a random intercept and slope for treatment effect by randomisation block and an additional random slope for treatment by intervention group cluster. In main analyses, in addition to a fixed effect for assignment to the intervention arm, we included a fixed effect for baseline score. The exception was for the SGLSS analysis immediately post-intervention for which we simplified the model to facilitate convergence (see Additional file 4: S4) and included random intercepts but not random slopes. In pre-specified adjusted analyses, we also controlled for age (years), sex (male versus female), whether the leader has SSc (yes versus no), and leader experience (existing versus candidate; for the PHQ-8 and OLBI only) as fixed effects.

To minimize the possibility of bias from missing outcome data, we used multiple imputation by chained equations using the mice package to generate 20 imputed datasets, using 15 cycles per imputed dataset. Variables included in the mice procedure are described in Additional file 4: S4. Pooled standard errors and associated 95% CIs were estimated using Rubin’s rules [80].

All analyses were 2-sided with α = 0.05. We did not adjust for multiple analyses since we identified a single primary outcome a priori. We did not report data separately by sex or race or ethnicity due to the small number of male participants and of participants who identified as Black, Hispanic or Latino, or another race or ethnicity.

### Protocol amendments

There were three amendments. First, in addition to recruiting participants affiliated with patient organizations from Canada, the United States, the United Kingdom, Australia, and New Zealand, we accepted participants affiliated with patient organizations from other countries. Second, the protocol indicated that training groups would include 6 participants, but for scheduling feasibility, we formed groups with 5 or 6 participants. Third, we discontinued recruitment prior to randomising our targeted number of 182 participants. When we ceased initiation of new trial waves, we had randomised all enrolled participants whose availability matched that of other potential participants. Since the proportion of participants who provided data post-intervention exceeded our assumptions, we successfully collected outcome data for at least 146 participants at each post-intervention assessment, which was our targeted number of participants with outcome data.

### Patient and public involvement

The SPIN Support Group Advisory Team was involved in all stages of SPIN research on support groups in SSc, including preliminary research on support groups in SSc; the development of the SPIN-SSLED program; the design, implementation, and reporting of the feasibility trial; and the design, implementation, and reporting of the present full-scale trial. Members of the Team initially participated in planning and reviewing qualitative interviews with support group leaders, members, and non-participants; followed by the design of the Scleroderma Support Group Survey, which informed program development by collecting information on the experiences and training needs of SSc support group leaders, priorities of SSc support group members, and reasons why people do not attend SSc support groups [10–12, 23, 24]. Team members participated in the development of the SGLSS, which was validated [48], administered in the feasibility trial [43], and was the primary outcome for the full-scale trial. Team members, along with patient organization leaders from Canada and the United States, met with the research team and provided input into the development of the SPIN-SSLED Program and its modules, filmed the vignettes used in the program, and were involved in decisions related to the design and conduct of the feasibility and full-scale trials. They were involved in interpretation of results and reviewed and provided comments on the present trial report.

## Results

### Participants

**Fig. 1:**
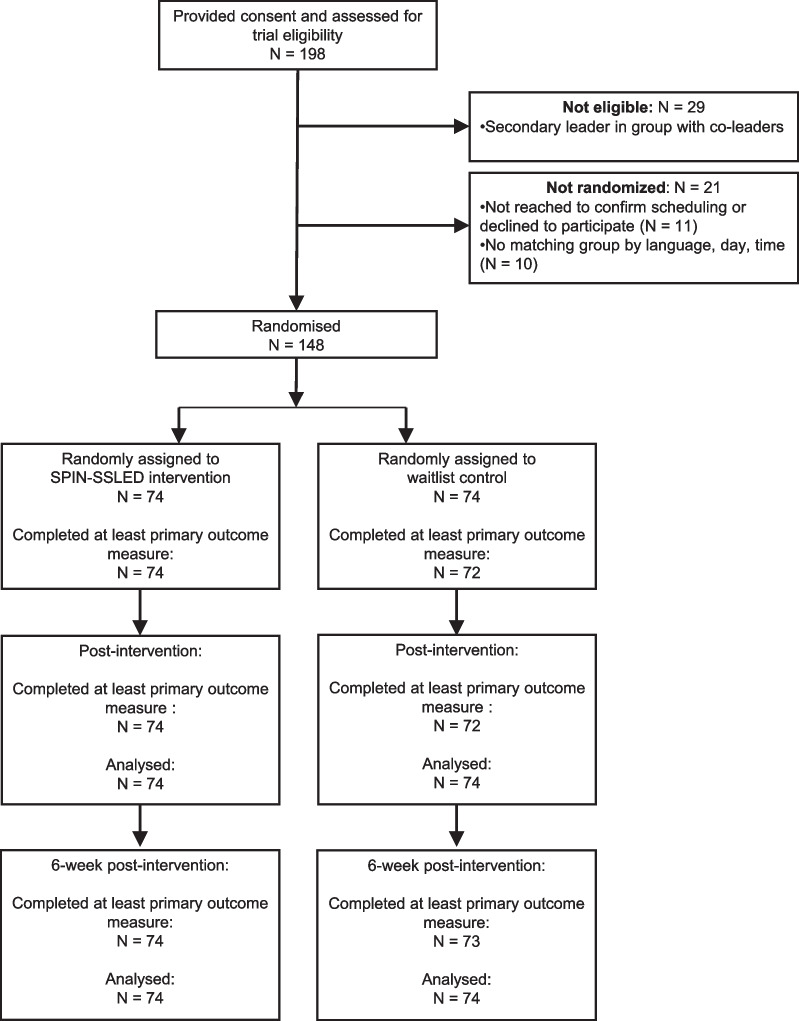
SPIN-SSLED trial flow diagram

A total of 198 potential participants were assessed for eligibility. Of these, 29 were not eligible because they were a co-leader of a group and registered as secondary to an eligible leader who was included. An additional 11 could not be reached to confirm scheduling or declined to participate, and 10 were not randomised due to the inability to match language or day and time availability. Thus, 148 participants were randomised to intervention (N = 74) or waitlist control (N = 74). See Fig. 1.

As shown in Table 2, intervention and waitlist participants were similar. Overall, among the 148 participants, mean age was 55.4 years (standard deviation [SD] = 11.9), 134 (91%) were female, 121 (of 146 who reported race or ethnicity; 83%) identified as White, and 130 had been diagnosed with SSc (88%). Participants were from the United States (N = 91, 61%), Canada (N = 22, 15%), and 7 other countries (N = 35, 24%). There were 114 (77%) experienced leaders with a mean of 5.2 years (SD = 5.6) leading a SSc support group.

**Table 2:** Participant characteristics

Variable	SPIN-SSLED intervention N = 74	Waitlist control N = 74
Demographics		
Female sex, N (%)	68 (92%)	66 (89%)
Age in years, mean (SD)	53.5 (12.0)	57.3 (11.7)
Education in years, mean (SD)	15.8 (2.6)	16.0 (3.1)
Live in a city or town (versus suburb or rural), N (%)	43 (58%)	40 (54%)
Married or living as married (versus single), N (%)	49 (66%)	51 (69%)
Work full-time or part-time, N (%)	30 (41%)	25 (34%)
Country, N (%)		
United States	43 (58%)	48 (65%)
Canada	13 (18%)	9 (12%)
Australia	5 (7%)	7 (9%)
New Zealand	4 (5%)	4 (5%)
United Kingdom	5 (7%)	1 (1%)
Other^a^	4 (5%)	5 (7%)
Language of instruction, N (%)^b^		
English	69 (93%)	69 (93%)
French	5 (7%)	5 (7%)
Race/ethnicity, N (%)^c,d^		
White	58 (79%)	63 (86%)
Black	4 (5%)	3 (4%)
Hispanic/Latino	5 (7%)	1 (1%)
Other^e^	6 (8%)	6 (8%)
Disease characteristics		
Diagnosed with SSc, N (%)	63 (85%)	67 (91%)
Diffuse disease subtype among participants diagnosed with SSc, N (%)	30 (48%)	26 (39%)
Years since SSc diagnosis among participants with SSc, mean (SD)^f^	12.4 (8.4)	13.5 (7.7)
Support group leader experience		
Experienced leader of SSc support group, n (%)	57 (77%)	57 (77%)
Years as a SSc support group leader among experienced leaders, mean (SD)^g^	4.6 (5.7)^h^	5.8 (5.6)^i^
Works with a co-leader among experienced leaders, n (%)	26 (49%)^h^	26 (46%)^i^
Former regular member of their support group among experienced leaders, n (%)	26 (49%)^h^	32 (57%)^i^
Patient-reported outcomes at baseline		
Scleroderma Support Group Leader Self-Efficacy Scale, mean (SD)	149.8 (23.0)	149.3 (20.9)^j^
Patient Health Questionnaire-8, mean (SD)	3.7 (3.3)	4.0 (3.9)^j^
Oldenburg Burnout Inventory, among experienced leaders, mean (SD)	38.6 (3.3)	38.6 (3.4)^k^
Volunteer Satisfaction Index, among experienced leaders, mean (SD)^l^	36.4 (7.2)	34.8 (8.1)^k^

*N* number of participants, *SD* standard deviation, *SSc* systemic sclerosis

^a^Philippines, N = 4; France, N = 3; Germany, N = 1; India, N = 1

^b^Based on expressed preference and intervention or waitlist assignment

^c^N respondents = 73 for intervention and waitlist arms

^d^Participants who provided more than one race/ethnicity were classified as other

^e^American Indian or Alaskan Native, N = 2; Asian, N = 4; Indo-Aryan, N = 1; Maori, N = 1; Mixed, N = 1; White and Aboriginal, N = 1; White and American Indian or Alaskan Native, N = 1; White and Asian, N = 1

^f^Maximum years a participant could enter was “25 or more”, which was counted as 25 years (N = 12 in intervention arm and 9 in waitlist arm)

^g^Maximum years a participant could enter was “25 or more”, which was counted as 25 (N = 2 in intervention arm and 0 in waitlist arm)

^h^N = 53 due to missing data for 4 participants

^i^N = 56 due to missing data for one participant

^j^N = 72 due to missing data for one participant

^k^N = 55 due to missing data for 2 participants

^l^For 16 participants in the intervention arm and 18 participants in the waitlist control arm, item 5 (of 7 items) was not administered due to a technical error; for those participants, total scores were calculated by multiplying total scores on 6 items administered by 7/6

### Intervention sessions

The 74 intervention participants were assigned to one of 13 groups (12 English, 1 French) in wave 1 (3 groups; September–December 2019), wave 2 (4 groups; January–March 2020), wave 3 (2 groups; April–June 2020), wave 4 (3 groups; July–September 2020), or wave 5 (1 group; January–March 2021). The mean number of sessions attended was 11.4 (SD = 2.6; median = 12); 2 (3%) participants enrolled but did not attend any sessions, 4 (5%) attended 3–8 sessions, and 68 (92%) attended 10–13 sessions. In the 41 sessions evaluated for program adherence, 466 of 468 (> 99%) session components were delivered as planned.

### Trial outcomes

Outcome data were obtained for 146 of 148 (99%) of participants immediately post-intervention, including 74 of 74 (100%) intervention participants and 72 of 74 (97%) waitlist participants. At 3 months post-intervention, 147 (99%) provided follow-up data, including 74 (100%) intervention participants and 73 (99%) waitlist participants. Table 3 shows complete-data outcomes at each time point.

**Table 3:** Outcome data immediately post-intervention and 3 months post-intervention (complete data only)

	SPIN-SSLED intervention		Waitlist control	
	N	Mean (SD)^a^	N	Mean (SD)^a^
*Post-intervention*				
Leader self-efficacy (SSGLSS) score	74	166.9 (13.8)	72	150.2 (21.3)
Emotional distress (PHQ-8) score	74	2.9 (3.0)	72	4.1 (4.0)
Burnout (OLBI) score (among experienced leaders)	56	37.9 (3.4)	54	38.6 (4.4)
Volunteer satisfaction (VSI) score (among experienced leaders)	56	40.9 (5.3)	54	35.0 (7.5)
*Months post-intervention*				
Leader self-efficacy (SSGLSS) score	73^b^	164.4 (15.1)^b^	73	148.4 (23.2)
Emotional distress (PHQ-8) score	74	3.4 (3.5)	72	4.5 (4.6)
Burnout (OLBI) score (among experienced leaders)	57	37.8 (3.0)	53	38.9 (3.8)
Volunteer satisfaction (VSI) score (among experienced leaders)	57	39.7 (6.0)	52	34.6 (7.9)

*N* number of participants, *PHQ-8* Patient Health Questionnaire-8, *OLBI* Oldenburg Burnout Inventory, *SD* standard deviation, *SSGLSS* Scleroderma Support Group Leader Self-efficacy Scale, *VSI* Volunteer Satisfaction Index

^a^Standard deviations do not take into account clustering within intervention groups

^b^One intervention arm participant scored 118 on the Scleroderma Support Group Leader Self-Efficacy Scale (possible scores 32–192) at baseline, 187 post-intervention, and 32 (all items “strongly disagree”) at 3 months post-intervention. We inquired with the participant about the unusual variability in scores, and she indicated that she had intended to score all items as “strongly agree” at 3 months post-intervention (score = 192) but had mistakenly responded backwards. Thus, her score was counted as missing at 3 months post-intervention

As shown in Table 4, in the primary intent-to-treat analysis, SGLSS scores were statistically significantly higher, reflecting greater self-efficacy, immediately post-intervention among intervention compared to waitlist participants (16.68 points, 95% CI 11.04–22.32; SMD = 0.84, 95% CI 0.58–1.09). They were also significantly higher at 3 months post-intervention (15.61 points, 95% CI 10.24–20.97; SMD = 0.73, 95% CI 0.49–0.98). SGLSS scores among waitlist participants were within one point of pre-intervention scores both post-intervention and 3 months later, whereas scores of intervention arm participants improved by 17 points post-intervention and were 15 points higher 3 months later compared to baseline. Emotional distress was significantly lower among intervention participants immediately post-intervention (− 0.93 points, 95% CI − 1.84 to − 0.03; SMD = − 0.26, 95% CI − 0.50 to − 0.02) but not 3 months post-intervention (− 0.81 points, 95% CI − 1.86 to 0.25 points; SMD = − 0.19, 95% CI − 0.41 to 0.02) compared to waitlist participants.

**Table 4:** Trial outcomes: Intent to treat and adjusted intent to treat

	Intent to treat^a^		Adjusted intention to treat^b^	
	Difference (95% CI)	Hedges’ g SMD (95% CI)	Difference (95% CI)	Hedges’ g SMD (95% CI)
*Primary outcome (post-intervention)*				
Leader self-efficacy (SSGLSS) score^c^	16.68 (11.04, 22.32)	0.84 (0.58, 1.09)	17.05 (11.81, 22.29)	0.85 (0.59, 1.11)
*Secondary outcomes (post-intervention)*				
Emotional distress (PHQ-8) score	− 0.93 (− 1.84, − 0.03)	− 0.26 (− 0.50, − 0.02)	− 1.15 (− 2.03, − 0.26)	− 0.32 (− 0.56, − 0.08)
Burnout (OLBI) score (among experienced leaders)	− 0.53 (− 1.66, 0.59)	− 0.13 (− 0.38, 0.11)	− 0.64 (− 1.81, 0.54)	− 0.16 (− 0.41, 0.09)
Volunteer satisfaction (VSI) score (among experienced leaders)	5.08 (3.25, 6.91)	0.70 (0.46, 0.94)	4.94 (3.05, 6.82)	0.68 (0.45, 0.92)
*Secondary outcomes (3 months post-intervention)*				
Leader self-efficacy (SSGLSS) score	15.61 (10.24, 20.97)^d^	0.73 (0.49, 0.98)^d^	16.20 (10.63, 21.78)^d^	0.76 (0.51, 1.01)^d^
Emotional distress (PHQ-8) score	− 0.81 (− 1.86, 0.25)	− 0.19 (− 0.41, 0.02)	− 1.01 (− 2.03, 0.00)	− 0.24 (− 0.46, − 0.03)^e^
Burnout (OLBI) score (among experienced leaders)	− 0.91 (− 1.94, 0.13)	− 0.25 (− 0.51, 0.00)	− 0.98 (− 2.05, 0.08)	− 0.28 (− 0.53, − 0.02)^e^
Volunteer satisfaction (VSI) score (among experienced leaders)	3.91 (2.10, 5.72)	0.53 (0.31, 0.75)	3.91 (2.09, 5.72)	0.53 (0.32, 0.75)

All models are presented with multiply imputed data. For the Scleroderma Support Group Leader Self-Efficacy Scale and Volunteer Satisfaction Index, positive numbers favour the intervention. For the Patient Health Questionnaire-8 and Oldenburg Burnout Inventory, negative numbers favour the intervention. Analyses of SSGLSS and PHQ-8 include all 148 participants, whereas analyses of OLBI and VSI include only the 114 experienced participants, since these are not relevant to candidate leaders

*CI* confidence interval, *PHQ-8* Patient Health Questionnaire-8, *OLBI* Oldenburg Burnout Inventory, *SMD* standardized mean difference, *SSGLSS* Scleroderma Support Group Leader Self-efficacy Scale, *VSI* Volunteer Satisfaction Index

^a^Adjusted for baseline outcome score only

^b^Adjusted for baseline score plus age (continuous); sex (male vs. female); whether the leader has scleroderma (no vs. yes); and, for outcomes that included both experienced and new leaders, whether the leader was a prospective vs. experienced leader

^c^Models were simplified to remove random slopes and to only include a single random intercept for each separate intervention group and for all waitlist participants combined to facilitate convergence

^d^One intervention arm participant scored 118 on the Scleroderma Support Group Leader Self-Efficacy Scale (possible scores 32–192) at baseline, 187 post-intervention, and 32 (all items “strongly disagree”) at 3 months post-intervention. We inquired with the participant about the unusual variability in scores, and she indicated that she had intended to score all items as “strongly agree” at 3 months post-intervention (score = 192) but had mistakenly responded backwards. Thus, her score was counted as missing at 3 months post-intervention

^e^The Borenstein and Hedges’ formula for converting regression coefficients to standardized mean differences was adapted for use with the PN-RCT design and, thus, closely approximates the SMD and 95% CI. In two cases, there were discrepancies between statistical significance based on raw scale scores and Hedges’ g; the raw score results should be interpreted for statistical significance. See Additional file 6: S6

Among leaders in the intervention arm who were leading groups at the start of the trial, satisfaction that their volunteer support group leader role was benefitting others was significantly higher immediately post-intervention (5.08 points, 95% CI 3.25–6.91; SMD = 0.70, 95% CI 0.46–0.94) and 3 months later (3.91 points, 95% CI 2.10–5.72; SMD = 0.53, 95% CI 0.31–0.75) compared to waitlist participants. Burnout was not significantly different between groups immediately post-intervention (− 0.53, 95% CI − 1.66 to 0.59; SMD = − 0.13, 95% CI − 0.38 to 0.11) or 3 months post-intervention (− 0.91, 95% CI − 1.94 to 0.13; SMD = − 0.25, 95% CI − 0.51 to 0.00). Results were similar for all outcomes in complete case analyses (Additional file 5: S5).

Among intervention participants who attended at least one training session (N = 72), satisfaction with the program was high. Mean CSQ-8 score was 30.3 (SD = 3.0) of a maximum total score of 32 points. Mean item score was 3.8 (possible 1–4). See Additional file 6: S6. No adverse events were reported by participants.

## Discussion

We tested whether a program developed in collaboration with scleroderma peer support group leaders and members of patient organization leadership teams would increase peer-leader self-efficacy, including confidence to perform organizational tasks, manage group and interpersonal interactions, and balance group leadership and self-care needs. In our primary analysis, leader self-efficacy scores were substantially higher (SMD = 0.84, 95% CI 0.58–1.09) immediately post-intervention among intervention versus waitlist participants. They were also higher 3 months later (SMD = 0.73, 95% CI 0.49–0.98). These are considered large effects based on common metrics. [81]

Among secondary outcomes, the intervention significantly reduced emotional distress and improved volunteer satisfaction, but not burnout, immediately post-intervention. At 3 months post-intervention, only volunteer satisfaction was significantly better in the intervention group. Mean increases in volunteer satisfaction at both time points are considered medium to large effects and the reduction in emotional distress immediately post-intervention a small effect [81].

Among participants in the intervention arm, over 90% attended at least 10 of 13 sessions, even though many participants were people with severe SSc; in some instances, participants logged into training sessions from the hospital. Participant satisfaction with the program was very high with an average item rating of 3.8 out of a maximum of 4 points on 8 items.

One previous randomised trial has tested an intervention that involved training, education, or support to peer support group leaders [42]. That was a small trial (N = 52) that assigned leaders of cancer support groups to a website and discussion forum or website and discussion forum plus 2 days workshop. No significant differences in leader self-confidence or self-efficacy were found. In addition to differences in trial methods and the number of participants enrolled, there were important differences in the SPIN-SSLED Program intervention compared to the intervention tested in that trial. First, intervention materials in the cancer support group leader trial were developed by experts based on evidence from a literature review of group facilitation practices and the cancer support group leader literature. The SPIN-SSLED Program was developed jointly by SSc patient organization leaders, a patient advisory team, and researchers based on evidence of best practices in group facilitation plus qualitative and survey research conducted over several years by the project team [10–12, 23, 24]. Modifications to SPIN-SSLED Program content to better address user needs were made based on recommendations from participants in a feasibility trial [43]. Second, training in the SPIN-SSLED Program occurred weekly over a 13 weeks period compared to a 2 days workshop in the previous trial. Third, to reach people across the world in a rare-disease context, the SPIN-SSLED Program was delivered via videoconference, rather than face-to-face. This provided an additional benefit of allowing the trial to continue without interruption during the COVID-19 pandemic.

Although the program was developed by and for people with SSc, it is a general support group leader program with only one module that is SSc-specific. The program can be easily adapted for support groups in other rare conditions by simply removing the single SSc-specific module or replacing it. To facilitate adaptation and uptake, SPIN has begun planning for work to determine how the program may be adapted to best serve the needs of other disease patient organizations. This will include consultations with SPIN-SSLED Program graduates to understand the most helpful aspects of the program, as well as components that might be improved, based on their experiences implementing program learning in their ongoing support groups. Additionally, we are consulting with the leadership of other patient organizations so that program adaptation recommendations fit their organizational needs and structure. Ideally, our trial will be replicated in one or more other settings and, if also found to be successful there, implemented more broadly. Based on the positive results of our trial, the SPIN-SSLED Program is currently being provided post-trial on an ongoing basis by SPIN and patient organization partners. Future trials may consider increasing the number of participants per group, which could reduce delivery cost per person without negatively influencing benefits. We have successfully included 8–10 participants in post-trial training groups that we are providing. All SPIN-SSLED Program materials are published under Creative Commons copyright licenses, which will facilitate use by others.

The SPIN-SSLED Trial had important strengths. The program was developed as a patient-health care professional collaboration over several years, based on extensive qualitative and survey research with support group leaders, attendees, and non-participants, and it was feasibility tested. The primary outcome measure was developed and validated to assess the training objectives of the program. We successfully recruited a large number of SSc support group leaders from 9 countries to participate in the trial, we met our recruitment target for the number of participants with outcome data, and we conducted the trial with careful attention to methodological and reporting standards. Adherence to the program among intervention participants was high, and we collected follow-up outcome data from 99% of participants both immediately post-intervention and 3 months later. The program is feasibly delivered via online videoconference at a low cost, which will support dissemination, and it is easily adapted for peer leaders of support groups for people with different medical conditions.

A limitation of the trial is that we assessed self-report outcomes from support group leaders, but we did not evaluate whether participation in the training program led to improvements in support group quality, satisfaction of support group participants, or sustainability of groups. The decision to focus on support group leaders was made based on two considerations. One was that we believed that attempting to incorporate these other aspects would have increased the complexity and cost of the trial substantially to the extent that the cost of such a trial would have been prohibitive. Second, while some support group participants attend the same group consistently, many others come and go. It would have been difficult to define the participant population, and we were concerned that we may not have been able to obtain sufficiently complete data to be confident in results. Self-efficacy is associated with improved outcomes among people with medical conditions and in training of health care providers and other educators [45–47], and, we believe that improved peer leader self-efficacy will similarly result in more effective peer support and education. Another limitation is that most trial participants were White, English-speaking, middle-aged, and women; it is possible that generalizability may be limited by this.

## Conclusions

Peer-led support groups are used by people with rare diseases around the world for emotional and practical support and education to strengthen coping skills. They are organized and delivered, in most cases, without collaboration from health care professionals. The SPIN-SSLED Trial is the first well-conducted, adequately powered trial in any disease to test whether a support group leader education and training program can improve support group leader self-efficacy for group leadership. The program led to substantially higher leader self-efficacy, and this was retained 3 months post-intervention. It also improved satisfaction of leaders with their volunteer contributions and may reduce emotional distress. The program is feasibly delivered via internet videoconference. Peer support group leader training may increase the likelihood that groups provide intended benefits and reduce the potential for harm through misinformation via modules that address finding credible information and the importance of sharing experience but not medical information or advice. Results from the SPIN-SSLED Trial should be replicated in other conditions and, if successful, the program can be adapted and disseminated to patient organizations that support people with other rare diseases.

## Supplementary Information

**Additional file1**. **S1**: Examples of support groups for people with systemic sclerosis.

**Additional file2**. **S2**: Links to SPIN-SSLED Participant Program Manual, Instructor Manual, and PowerPoint slides in English and French.

**Additional file3**. **S3**: Detailed trial outcome measures.

**Additional file4**. **S4**: Statistical analysis plan.

**Additional file5**. **S5**: Trial outcomes for intent to treat and adjusted intent to treat with complete cases only (no imputation).

**Additional file6**. **S6**: Client Satisfaction Questionnaire-8 item and total scores.

## Data Availability

De-identified individual participant data with a data dictionary and analysis codes that were used to generate the results reported in this article will be made available upon request to the corresponding author and presentation of a methodologically sound proposal that is approved by the Scleroderma Patient-centered Intervention Network Data Access and Publications Committee. Data will be available beginning 12 months after publication. Data requesters will need to sign a data transfer agreement. All materials used in the study are copyrighted with Creative Commons licenses with links in Additional file 2: S2.

## Abbreviations

CI: Confidence interval
CONSORT: Consolidated Standard of Reporting Trials
CSQ-8: Client Satisfaction Questionnaire-8
ICC: Intra-class correlation coefficient
OLBI: Oldenburg Burnout Inventory
PHQ-8: Patient Health Questionnaire-8
PN-RCT: Partially nested randomized controlled trial
SGLSS: Support Group Leader Self-efficacy Scale
SMD: Standardized mean difference
SPIN: Scleroderma Patient-centered Intervention Network
SSLED: Scleroderma Support Group Leader Education
SSc: Systemic sclerosis
VSI: Volunteer Satisfaction Index
