# Supplementary material for: Effects of a support group leader education program jointly developed by health professionals and patients on peer leader self-efficacy among leaders of scleroderma support groups: a two-arm parallel partially nested randomised controlled trial
Source: Orphanet J Rare Dis. 2022 Oct 28;17:396. doi: 10.1186/s13023-022-02552-x (PMC9616616; doi:10.1186/s13023-022-02552-x)
Supplement: Supplementary file 1 — Additional file1. S1: Examples of support groups for people with systemic sclerosis. [file 13023_2022_2552_MOESM1_ESM.docx]

**Supplementary Material 1.** Examples of support groups for people with systemic sclerosis

| **Country** | **Organization** | **Links (Accessed June 5, 2022)** |
| --- | --- | --- |
| Australia | Scleroderma Australia | https://www.sclerodermaaustralia.com.au/get-support/ |
| Australia | Scleroderma Association of New South Wales | https://www.sclerodermansw.org/ |
| Australia | Scleroderma Queensland | https://www.scleroderma.org.au/ |
| Australia | Scleroderma Victoria | https://sclerodermavictoria.com.au/ |
| Belgium | Association des Patients Sclérodermiques de Belgique | https://www.sclerodermie.be |
| Canada | Scleroderma Canada | https://www.scleroderma.ca/copy-of-patient-support |
| Canada | Scleroderma Alberta | https://www.facebook.com/Scleroderma-Alberta-546840705399094/ |
| Canada | Scleroderma Association B.C. | https://sclerodermabc.ca/support-and-assistance/find-a-support-group/ |
| Canada | Scleroderma Atlantic | https://www.sclerodermaatlantic.ca/about-scleroderma-atlantic/ |
| Canada | Scleroderma Society of Ontario | https://www.hardword.ca/patient-support |
| Canada | Scleroderma Manitoba | https://sclerodermamanitoba.com/support-in-your-community/ |
| Canada | Sclérodermie Québec | https://sclerodermie.ca/en/support-and-assistance/ |
| Canada | Scleroderma SASK | https://sclerodermasaskatchewan.ca/ |
| Czech Republic | Revma Liga Česká republika | https://www.revmaliga.cz/kluby/ |
| Denmark | Sklerodermiforeningen | https://sklerodermi.dk/nordjylland/ |
| France | Association des Sclérodermiques de France | https://www.association-sclerodermie.fr/association/actions/#rencontres |
| New Zealand | Scleroderma New Zealand Inc. | https://scleroderma.org.nz |
| Spain | Associación Española Esclerodermia | https://esclerodermia.com/grupo-de-apoyo-emocional/ |
| Switzerland | Sclerodermie.ch | https://sclerodermie.ch |
| United Kingdom | Scleroderma and Raynaud's UK | https://www.sruk.co.uk/find-support/support-groups/ |
| United States | National Scleroderma Foundation through local and state chapters | https://scleroderma.org/scleroderma-support-groups/ |
