# Supplementary material for: Effects of a support group leader education program jointly developed by health professionals and patients on peer leader self-efficacy among leaders of scleroderma support groups: a two-arm parallel partially nested randomised controlled trial
Source: Orphanet J Rare Dis. 2022 Oct 28;17:396. doi: 10.1186/s13023-022-02552-x (PMC9616616; doi:10.1186/s13023-022-02552-x)
Supplement: Supplementary file 2 — Additional file2. S2: Links to SPIN-SSLED Participant Program Manual, Instructor Manual, and PowerPoint slides in English and French. [file 13023_2022_2552_MOESM2_ESM.docx]

**Supplementary Material 2.** Links to SPIN-SSLED Participant Program and Instructor Material

Participant manuals, instructor manuals, and module PowerPoint slides in English and French can be accessed via the SPIN-SSLED Open Science Framework page (<https://osf.io/dwz5c/>).
