# Supplementary material for: Effects of a support group leader education program jointly developed by health professionals and patients on peer leader self-efficacy among leaders of scleroderma support groups: a two-arm parallel partially nested randomised controlled trial
Source: Orphanet J Rare Dis. 2022 Oct 28;17:396. doi: 10.1186/s13023-022-02552-x (PMC9616616; doi:10.1186/s13023-022-02552-x)
Supplement: Supplementary file 3 — Additional file3. S3: Detailed trial outcome measures. [file 13023_2022_2552_MOESM3_ESM.docx]

**Supplementary Material 3.** Detailed trial outcome measures

The primary outcome analysis compared Support Group Leader Self-efficacy Scale (SGLSS)^1^ scores between group leaders allocated to the SPIN-SSLED Program versus the waitlist control immediately post-intervention. The SGLSS is a 32-item scale designed to assess SSc support group leader confidence to successfully perform leadership tasks (e.g., organizational skills), manage group and interpersonal interactions, and balance group leadership and self-care needs. The measure reflects the core educational content of the SPIN-SSLED Program. It utilizes a 6-point Likert scale ranging from 1 (strongly disagree) to 6 (strongly agree), with higher total scores (possible range 32 to 192) indicating greater self-efficacy. Prior to the development of the SGLSS, there were no existing measures of support group leader self-efficacy validated in any medical condition. We developed the SGLSS with our Support Group Leader Advisory Team based on evidence from systemic sclerosis (SSc) support group leader, support group participant, and support group non-participant interviews and surveys,^2-5^ translated it into French using forward-backward translation,^6^ and validated it in two samples of SSc support group leaders (N = 102, N = 55).^1^ We found that it had good internal consistency (Cronbach’s alpha 0.96 and 0.95) and hypothesis-consistent convergent validity with a burnout measure, the Oldenburg Burnout Inventory (OLBI).^7^ In the SPIN-SSLED Feasibility Trial,^8^ SGLSS pre-post difference was large among participants (standardized mean difference = 1.7; 1.1 point difference per item), suggesting sensitivity to change. Cronbach’s alpha was 0.96 among 146 participants who completed the SGLSS at baseline in the SPIN-SSLED Trial.

Secondary outcomes included the SGLSS 3 months post-intervention and emotional distress, measured by the Patient Health Questionnaire-8 (PHQ-8);^9^ burnout, measured by the OLBI;^7^ and leader satisfaction, measured by the Participation Efficacy subscale of the Volunteer Satisfaction Index (VSI)^10^ immediately post-intervention and 3 months post-intervention. The OLBI and VSI were only administered to existing but not candidate support group leaders because they evaluate experiences related to leading a support group. We additionally evaluated participant satisfaction among all participants randomised to the intervention via the Client Satisfaction Questionnaire-8 (CSQ-8)^11^ immediately post-intervention.

Emotional distress was assessed with the PHQ-8.^9^ The 8 items of the PHQ-8 measure depression symptoms over the last 2 weeks on a 4-point scale, ranging from 0 (not at all) to 3 (nearly every day). Item scores are summed to a total score with higher scores (possible range 0 to 24) indicating more depression symptoms. The PHQ-8 performs equivalently to the PHQ-9,^12^ which is a valid measure of depression symptoms in people with SSc.^13^ The PHQ-8 is available in French and English.^14^ The PHQ-8 is commonly used instead of the PHQ-9, because the PHQ-9 includes an item that includes both thoughts of death and thoughts of self-harm, which has been shown to elicit a high proportion of false-positive responses among people with medical illnesses but contribute inconsequentially to the validity of symptom measurement.^12^ Cronbach’s alpha for the baseline PHQ-8 assessment in the SPIN-SSLED Trial was 0.82 (N = 146).

Leader burnout was measured by the 16-item OLBI,^7^ which assesses current exhaustion and disengagement due to burnout and has been validated in diverse populations via positively and negatively worded items (4-point scale from 1 = strongly disagree to 4 = strongly agree).^7,15^ Item scores are summed to calculate the total scale score (items 2, 3, 4, 6, 8, 9, 11, 12 reverse scored). Total possible scores range from 16 to 64, and higher scores indicate higher levels of burnout. The OLBI was initially designed for work-related burnout but has been adapted for numerous settings and in multiple countries and languages.^16^ The SPIN Team revised the wording of each of the OLBI items in the English and French^17^ versions to reflect the support group environment rather than a work environment (e.g., “I find my work to be a positive challenge” was revised to “I find my role as a support group leader to be a positive challenge”). The OLBI has been validated in multiple populations and has good measurement properties.^7,15,16^ Internal consistency reliability (Cronbach’s alpha) in a previous study of 102 SSc support group leaders was 0.84 for exhaustion and 0.80 for disengagement.^1^ In the present trial, it was 0.86 for exhaustion and 0.84 for disengagement among the 112 experienced leaders who completed the OLBI at baseline.

Leader satisfaction (participation efficacy) was measured using a modified version of the participation efficacy subscale of the VSI.^10^ The original version of the VSI was validated using a sample of volunteers (N = 327) and was found to be reliable and constructually valid.^10^ As other studies have done previously,^18,19^ we modified the wording of some of the items to reflect participants’ volunteer role as support group leaders. The participation efficacy subscale asks respondents to indicate their level of satisfaction on 7 items using a 7-point Likert scale from 1 (very dissatisfied) to 7 (very satisfied). Higher scores (possible range 7 to 49) reflect greater satisfaction. Cronbach’s alpha was 0.90 at baseline in the SPIN-SSLED Trial among the 78 participants with all items administered.

Participant satisfaction with the SPIN-SSLED Program among those allocated to the training program was evaluated with the CSQ-8,^11^ a standardized measure that is used to assess satisfaction with health services. Items are scored from 1 to 4, and different items have different response options. Items 1, 3, 6, and 7 are reverse scored. Total scores range from 8 to 32 with higher scores representing greater satisfaction. The CSQ-8 has been widely validated across a range of populations^11^ and is available in French. Cronbach’s alpha was 0.86 among the 72 participants who attended at least one SPIN-SSLED session.

**Supplementary Material 3 References**

1. Pal NE, Gumuchian ST, Delisle VC, et al. Development and preliminary validation of the Scleroderma Support Group Leader Self-efficacy Scale. *J Scleroderma Relat Disord*. 2018;3:106-11.
2. Gumuchian ST, Delisle VC, Kwakkenbos L, et al. Reasons for attending support groups and organizational preferences: the European scleroderma support group members survey. *Disabil Rehabil*. 2019;41:974-82.
3. Delisle VC, Gumuchian ST, Pelaez S, et al. Reasons for non-participation in scleroderma support groups. *Clin Exp Rheumatol*. 2016;34 Suppl 100:56-62.
4. Gumuchian ST, Delisle VC, Peláez S, et al. Reasons for not participating in scleroderma patient support groups: a cross-sectional study. *Arthritis Care Res*. 2018;70:275-83.
5. Delisle VC, Gumuchian ST, El-Baalbaki G, et al. Training and support needs of scleroderma support group facilitators: the North American Scleroderma Support Group Facilitators Survey. *Disabil Rehabil*. 2019:41:2477-82.
6. World Health Organization. WHODAS 2.0 translation guidelines. https://terrance.who.int/mediacentre/data/WHODAS/Guidelines/WHODAS%202.0%20Translation%20guidelines.pdf. Accessed September 15, 2020.
7. Halbesleben JRB, Demerouti E. The construct validity of an alternative measure of burnout: Investigating the English translation of the Oldenburg Burnout Inventory. *Work Stress*. 2005;19:208-20.
8. Thombs BD, Dyas L, Pépin M, et al. Scleroderma Patient-centered Intervention Network Support group Leader EDucation (SPIN-SSLED) Program: non-randomised feasibility trial. *BMJ Open*. 2019;9:e029935.
9. Kroenke K, Strine TW, Spitzer RL, et al. The PHQ-8 as a measure of current depression in the general population. *J Affect Disord*. 2009;114:163-73.
10. Galindo-Kuhn R, Guzley RM. The Volunteer Satisfaction Index. *J Soc Serv Res*. 2002;28:45-68.
11. Kelly PJ, Kyngdon F, Ingram I, et al. The Client Satisfaction Questionnaire-8: psychometric properties in a cross-sectional survey of people attending residential substance abuse treatment. *Drug Alcohol Rev*. 2018;37:79-86.
12. Wu Y, Levis B, Riehm KE, et al. Equivalency of the diagnostic accuracy of the PHQ-8 and PHQ-9: a systematic review and individual participant data meta-analysis. *Psychol Med*. 2020;50:1368-1380.
13. Milette K, Hudson M, Baron M, et al. Comparison of the PHQ-9 and CES-D depression scales in systemic sclerosis: internal consistency reliability, convergent validity and clinical correlates. *Rheumatology*. 2010;49:789-796.
14. Arthurs E, Steele RJ, Hudson M, et al. Are scores on English and French versions of the PHQ-9 comparable? An assessment of differential item functioning. *PLoS ONE*. 2012;7:e52028.
15. Demerouti E, Bakker AB, Vardakou I, Kantas A. The convergent validity of two burnout instruments: a multitrait-multimethod analysis. *Eur J Psychol Assess*. 2002;19:12-23.
16. Reis D, Xanthopoulou D, Tsaousis I. Measuring job and academic burnout with the Oldenburg Burnout Inventory (OLBI): factorial invariance across countries and samples. *Burn Res.* 2015;2:8-18.
17. Chevrier N. Adaptation Québécoise de l'Oldenberg Burnout Inventory (OLBI) [Quebec adaptation of the Oldenburg Burnout Inventory (OLBI)] Montreal, QC: Université du Québec; 2009.
18. Boezeman EJ, Ellemers N. Volunteer recruitment: the role of organizational support and anticipated respect in non-volunteers' attraction to charitable volunteer organizations. *J Appl Psychol*. 2008;93:1013-26.
19. Boezeman EJ, Ellemers N. Pride and respect in volunteers' organizational commitment. *Eur J Soc Psychol*. 2008;38:159-72.
