# Supplementary material for: Effects of a support group leader education program jointly developed by health professionals and patients on peer leader self-efficacy among leaders of scleroderma support groups: a two-arm parallel partially nested randomised controlled trial
Source: Orphanet J Rare Dis. 2022 Oct 28;17:396. doi: 10.1186/s13023-022-02552-x (PMC9616616; doi:10.1186/s13023-022-02552-x)
Supplement: Supplementary file 4 — Additional file4. S4: Statistical analysis plan. [file 13023_2022_2552_MOESM4_ESM.docx]

**Supplementary Material 4.** Statistical analysis plan

**STATISTICAL ANALYSIS PLAN (SAP) for the Scleroderma Patient-centered Intervention Network Support Group Leader Education (SPIN-SSSLED) Program Trial**

**ADMINISTRATIVE INFORMATION**

**Registered at:** https://clinicaltrials.gov/ct2/show/NCT03965780

**Protocol available at:** https://trialsjournal.biomedcentral.com/articles/10.1186/s13063-019-3747-z

**SAP Version 1 (2021-06-14):**

**SAP contributors**:

1. Brooke Levis (Keele University, UK)
2. Alexander W. Levis (Harvard University, USA)
3. Linda Kwakkenbos (Radboud University, the Netherlands)
4. Robert Platt (McGill University, Canada)
5. Andrea Benedetti (McGill University, Canada)
6. Brett Thombs (McGill University, Canada)

This SAP was drafted by Drs. Brooke Levis and Brett Thombs. All other contributors provided input and reviewed and approved the final version. Drs. Robert Platt and Andrea Benedetti are the senior statisticans responsible for the integrity of all analyses. Dr. Brett Thombs is the principal investigator and clinical lead.

**SAP Version 1 (2021-06-14)**

**Study Design**

The SPIN-SSLED Trial is a pragmatic, two-arm, parallel, blocked, partially nested randomised controlled trial (PN-RCT). The trial is a blocked PN-RCT because eligible participants will first be grouped into blocks (referred to as “pools” in main text) based on language and scheduling availability, and then randomised into intervention arm. Within each block, participants randomised to the intervention arm will interact with their group facilitator and with others in their group during videoconference sessions. Within each block, participants randomised to the waitlist control arm will not interact with each other and will only complete trial measures.

**Study Outcomes**

The primary outcome analysis will compare self-efficacy (Scleroderma Support Group Leader Self-efficacy Scale [SGLSS]) scores between participants randomly assigned to the SPIN-SSLED Program versus the waitlist control at the end of the 13-week intervention period.

Secondary outcomes will include (1) SGLSS scores 3 months post-intervention; (2) burnout, measured by the Oldenburg Burnout Inventory (OLBI) post-intervention and 3 months post-intervention; (3) leader satisfaction that leading a support group is helping others, measured by the Participation Efficacy subscale of the Volunteer Satisfaction Index (VSI) post-intervention and 3 months post-intervention; and (4) emotional distress, measured by the Patient Health Questionnaire-8 (PHQ-8) post-intervention and 3 months post-intervention.

**Sample Size**

We identified several meta-analyses that have evaluated self-efficacy in terms of knowledge acquisition and confidence in implementing skills acquired in training programs. A 2016 Cochrane review reported a standardized mean difference effect size of 0.87 for 4 educational interventions designed to change knowledge of sickle cell disease among patients and caregivers (standardized mean difference = 1.12 with an outlier study removed).^1^ Several other similarly focused meta-analyses have reported effect sizes of between 0.58 and 0.94.^2-5^ In the SPIN-SSLED non-randomised feasibility trial, which only included 10 participants, the pre-post change in self-efficacy for carrying out leadership tasks was 1.7 standard deviations.^6^ For an assumed effect size of 0.70, a two-tailed test with α = 0.05, and an intra-class correlation coefficient (ICC) of 0.05, N = 75 would provide ≥ 80% power for self-efficacy for carrying out leader tasks. There was no loss to follow-up in our feasibility trial. Assuming 20% loss to follow-up in the proposed trial, we would need to randomise 94 support group leaders. We believe that this is a conservative power and sample size estimate. First, based on existing systematic reviews and on results of our feasibility trial, we believe that the true effect size is likely larger than 0.70. Second, in cluster RCTs, ICC values for individual patient outcomes are typically lower than our 0.05 estimate, even when different interveners are involved,^7-9^ and we will use the same trainer across groups in each language. If the true ICC is lower than our 0.05 estimate, this will result in greater power than estimated. Third, there was no loss to follow-up in our feasibility trial, and in our previous completed studies in SSc that required follow-up, loss to follow-up has been 10% or less.^10,11^

For the secondary outcomes (emotional distress, burnout, leader satisfaction (participation efficacy)), based on published meta-analyses, a standardized mean difference effect size of 0.50 represents a clinically meaningful effect size for improvement that has been achieved in training programs for managers, caregivers of chronically ill patients, and parents of children with difficult behavior.^2,12-14^ This is also considered a clinically meaningful effect size for patient-reported health outcomes, including depressive symptoms.^15^ For effect size = 0.50, a two-tailed test with α = 0.05, and an ICC of 0.05, N = 146 would provide ≥ 80% power for both self-efficacy and patient-reported health outcomes. Assuming 20% loss to follow-up in the proposed trial, we would need to randomise 182 support group leaders. We did not estimate power for leader satisfaction.

Members of our Support Group Leader Advisory Team and our patient organization partners have emphasized the importance of evaluating the trial’s planned secondary outcomes. Thus, we will attempt to enrol 180 participants total (15 training groups of 6 participants; 90 participants in waitlist control) in order to have sufficient power to adequately evaluate secondary outcomes.

**Data Preparation and Analysis**

1. Data will be checked for completeness and cleaned
2. Descriptive Analyses:

- By arm (intervention vs waitlist), we will compare demographic and disease characteristics (see draft Table below)
- Participant reported satisfaction with the SPIN-SSLED Program post-intervention. The Client Satisfaction Questionnaire (CSQ-8) is an 8-item questionnaire evaluating user satisfaction. Items are scored on a 4-point Likert scale. Total scores range from 8 to 32, with higher scores indicating higher satisfaction with the service. Items have been modified slightly to refer to the SPIN-SSLED Program, as opposed to a generic service.

| **Variable** | **SPIN-SSLED Intervention** | **Waitlist** |
| --- | --- | --- |
| **Demographic** |  |  |
| Female sex, N (%) |  |  |
| Age in years, mean (SD) |  |  |
| Education in years, mean (SD) |  |  |
| Live in a city or town (versus suburb or rural), N (%) |  |  |
| Married or living as married (versus single), N (%) |  |  |
| Working full-time or part-time, N (%) |  |  |
| Country, N (%) |  |  |
| United States |  |  |
| Canada |  |  |
| Australia |  |  |
| New Zealand |  |  |
| United Kingdom |  |  |
| Other |  |  |
| Language of instruction, N (%) |  |  |
| English |  |  |
| French |  |  |
| Race/ethnicity, N (%) |  |  |
| White |  |  |
| Black |  |  |
| Hispanic/Latino |  |  |
| Other |  |  |
| **Disease characteristics** |  |  |
| Diagnosed with SSc, N (%) |  |  |
| Diffuse disease subtype among participants with SSc, N (%) |  |  |
| Years since SSc diagnosis among participants with SSc, mean (SD) |  |  |
| **Support group leader experience** |  |  |
| Experienced^a^ leader of SSc support group, N (%) |  |  |
| Years as a SSc support group leader among experienced leaders, mean (SD) (SD) |  |  |
| Works with a co-leader among experienced leaders, N (%) |  |  |
| Former regular member of their support group among experienced leaders, N (%) |  |  |
| **Patient-reported outcomes (baseline)** |  |  |
| Scleroderma Support Group Leader Self-Efficacy Scale, mean (SD) |  |  |
| Patient Health Questionnaire-8, mean (SD) |  |  |
| Oldenburg Burnout Inventory, among current leaders, mean (SD) |  |  |
| Volunteer Satisfaction Index, among current leaders, mean (SD) |  |  |

N = number of participants; SD = standard deviation; SSc = systemic sclerosis.

^a^Experienced leaders are participants who were already leading a support group at the time of trial enrolment.

1. Trial outcomes will be assessed pre-randomisation, immediately following the 13-week intervention, and 3 months after the end of the intervention period for both participants randomised to the intervention and participants randomised to waitlist.

All analyses will be conducted in R, and will be 2-sided, using an alpha value of 0.05. There will be no adjustment for the multiplicity of secondary outcomes.

For the primary outcome analysis (SGLSS post-intervention), we will use an intent-to-treat analysis that compares all participants randomly assigned to the SPIN-SSLED Program to all participants randomly assigned to the waitlist control. The intervention effect immediately post-intervention will be estimated using a linear mixed-effects model (using the lmer function in lme4),^16^ including baseline SGLSS scores as a fixed effect. To account for the clustering inherent to the blocked PN-RCT design, we will fit a random intercept and slope for intervention by randomisation block and an additional random slope for intervention-by-intervention cluster (i.e., participants in the intervention arm who were in a trial group with the same group leader).^17^

We will account for missing data by using multiple imputation by chained equations, using the mice package.^18^ We will generate 20 imputed datasets, using 15 cycles per dataset. Variables in the mice procedure will include: randomisation block, trial arm, number of intervention sessions attended, measures of all primary and secondary outcomes at all three assessment time points (baseline, post-intervention, and 3-months post-intervention), age, sex (female vs. male), years of education, living in a city or town (vs. suburb or rural), married or living as married vs. single, currently working full-time or part-time (vs. not currently working), Country (USA, Canada, UK, New Zealand, Australia, or Other), language of instruction (English vs. French), race/ethnicity (White, Black, Hispanic/Latino, or Other), whether the leader has SSc, diffuse vs. limited disease subtype (among those with SSc only), years since SSc diagnosis (among those with SSc only), whether the leader is an experienced (i.e., already leading a support group at the time of trial enrolment) vs. candidate (i.e., identified by a partner organization as intending to initiate a new support group) leader, years as a SSc support group leader (among experienced leaders only), whether leader works with a co-leader (among experienced leaders only), and whether the leader was formerly a member of their support group (among experienced leaders only). We will account for non-applicable responses (e.g., SSc related variables among participants without SSc) by using the “where” option in the mice package. For all outcome measures, we will use total scores rather than item scores, with one exception; for VSI scores at baseline, we will use the 7 items separately. This is because, due to a programming error at the beginning of the trial, several participants only completed 6 of the 7 items. Pooled standard errors and associated confidence intervals will be estimated using Rubin’s rules.^19^

1. As a secondary analysis of the SGLSS, we will also control for age (years), sex (male vs. female), whether the leader has SSc (no vs. yes), and whether the leader is an experienced leader candidate leader as fixed effects.
2. Analyses of secondary outcomes will similarly be done (1) controlling for baseline scores only and (2) controlling for baseline scores and additional covariates. However, OLBI and VSI were only collected and will only be assessed among experienced leaders, since they are not relevant to candidate leaders. Thus, these analyses will not include leader experience as an additional covariate.
3. To aid in the interpretation of study results, for primary and secondary outcomes, post-intervention and 3 months post-intervention, we will produce model-adjusted standardized mean differences (Hedges’ g), using an adapted version of the method proposed by Borenstein and Hedges.^20^ For each outcome and time point, we will calculate the associated Hedges’ g estimate by dividing the unstandardized regression coefficient for the intervention effect by the unadjusted standard deviation of the outcome variable. To produce standard errors, we will use formula 11.31 from Borenstein and Hedges, which incorporates an estimate of the proportion of unexplained variance in the model (i.e., “1 - R^2^” in a standard linear regression model).^20^ In our case, we will estimate this proportion by dividing the estimated residual variance from the model by the unadjusted variance of the outcome variable. Estimated Hedges’ g and associated standard errors will be computed in each imputed dataset and then results will be pooled across imputed datasets using Rubin’s rules.^19^ Since the Borenstein and Hedges formula was not designed for the PN-RCT but was adapted, it approximates Hedges’ g and 95% CI.

**Amendments to Statistical Analaysis Plan**

1. The mice procedure needed to be modified to run successfully. We removed randomisation block, intervention arm, whether the leader has SSc, and whether the leader is an experienced versus candidate leader from the list of variables included in the mice procedure, as these variables were collinear with other variables included in the mice procedure. We also did not use the ‘where’ option to account for non-applicable responses, as this was not possible after removing these variables from the mice procedure. Instead, we re-set non-applicable values to ‘NA’ after the mice procedure was completed.
2. For the primary outcome (SGLSS scores immediately post-intervention), the intended analytical models did not converge. Thus, these analysis models were simplified to remove random slopes and to only include a single random intercept for each separate intervention group and for all waitlist participants combined (i.e., not separated by randomisation block) to facilitate convergence.

**Supplementary Material 4 References**

Asnani MR, Quimby KR, Bennett NR, Francis DK. Interventions for patients and caregivers to improve knowledge of sickle cell disease and recognition of its related complications. *Cochrane Database Syst Rev*. 2016;10:CD011175.

1. Kaminski JW, Valle LA, Filene JH, Boyle CL. A meta-analytic review of components associated with parent training program effectiveness. *J Abnorm Child Psychol*. 2008;36:567-89.
2. Armour TA, Norris SL, Jack L, Jr., Zhang X, Fisher L. The effectiveness of family interventions in people with diabetes mellitus: a systematic review. *Diabet Med*. 2005;22:1295-305.
3. Jiang X, Wang J, Lu Y, Jiang H, Li M. Self-efficacy-focused education in persons with diabetes: a systematic review and meta-analysis. *Psychol Res Behav Manag*. 2019;12:67-79.
4. Zhao FF, Suhonen R, Koskinen S, Leino-Kilpi H. Theory-based self-management educational interventions on patients with type 2 diabetes: a systematic review and meta-analysis of randomized controlled trials. *J Adv Nurs*. 2017;73:812-33.

Thombs BD, Dyas L, Pépin M, et al. The Scleroderma Patient-centered Intervention Network - Scleroderma Support group Leader Eductation (SPIN-SSLED) Program: non-randomised reasibility trial. *BMJ Open*. 2019;9:e029935.

Taljaard M, Donner A, Villar J, et al. Intracluster correlation coefficients from the 2005 WHO Global Survey on Maternal and Perinatal Health: implications for implementation research. *Paediatr Perinat Epidemiol*. 2008;22:117-25.

Smeeth L, Ng ES. Intraclass correlation coefficients for cluster randomized trials in primary care: data from the MRC Trial of the Assessment and Management of Older People in the Community. *Control Clin Trials*. 2002;23:409-21.

Hannan PJ, Murray DM, Jacobs DR, Jr., McGovern PG. Parameters to aid in the design and analysis of community trials: intraclass correlations from the Minnesota Heart Health Program. *Epidemiology*. 1994;5:88-95.

Thombs BD, Jewett LR, Kwakkenbos L, Hudson M, Baron M, Canadian Scleroderma Research G. Major depression diagnoses among patients with systemic sclerosis: baseline and one-month followup. *Arthritis Care Res*. 2015;67:411-6.

Jewett LR, Razykov I, Hudson M, Baron M, Thombs BD, Canadian Scleroderma Research G. Prevalence of current, 12-month and lifetime major depressive disorder among patients with systemic sclerosis. *Rheumatology*. 2013;52:669-75.

Collins DB, Holton Iii EF. The effectiveness of managerial leadership development programs: A meta‐analysis of studies from 1982 to 2001. *Human Resour Dev Q*. 2004;15:217-48.

Lundahl B, Risser HJ, Lovejoy MC. A meta-analysis of parent training: moderators and follow-up effects. *Clin Psychol Rev*. 2006;26:86-104.

Sörensen S, Pinquart M, Duberstein P. How effective are interventions with caregivers? An updated meta-analysis. *Gerontologist*. 2002;42:356-72.

National Institute for Clinical E, Britain G. Depression: management of depression in primary and secondary care: National Institute for Clinical Excellence; 2004.

Bates D, Machler M, Bolker B, Walker S. Fitting linear mixed-effects models using lme4. J *Stat Softw*. 2015;67:1-48.

Lohr S, Schochet PZ, Sanders E. Partially Nested Randomized Controlled Trials in Education Research: A Guide to Design and Analysis. NCER 2014-2000. National Center for Education Research. 2014.

van Buuren S, Groothuis-Oudshoorn K. mice: Multivariate Imputation by Chained Equations in R. *J Stat Softw*. 2011;45:1-67.

Rubin DB. Multiple imputation for nonresponse in surveys. 1987 New York, NY: Wiley.

Borenstein M and Hedges LV. Chapter 11: Effect sizes for meta-analysis. In: Cooper H, Hedges LV, and Valentine JC, eds. The Handbook of Research Synthesis and Meta-analysis. 3^rd^ Edition. New York, NY: Russell Sage Foundation; 2019.
