# Supplementary material for: Effects of a support group leader education program jointly developed by health professionals and patients on peer leader self-efficacy among leaders of scleroderma support groups: a two-arm parallel partially nested randomised controlled trial
Source: Orphanet J Rare Dis. 2022 Oct 28;17:396. doi: 10.1186/s13023-022-02552-x (PMC9616616; doi:10.1186/s13023-022-02552-x)
Supplement: Supplementary file 5 — Additional file5. S5: Trial outcomes for intent to treat and adjusted intent to treat with complete cases only (no imputation). [file 13023_2022_2552_MOESM5_ESM.docx]

**Supplementary Material 5.** Trial outcomes for intent to treat and adjusted intent to treat with complete cases only (no imputation)^a^

|  |  | **Intent to Treat^b^** | **Adjusted Intention to Treat^c^** |
| --- | --- | --- | --- |
|  | **N**  **Analysed** | **Difference (95% CI)** | **Difference (95% CI)** |
| **Primary Outcome (post-intervention):** |  |  |  |
| Leader self-efficacy (SGLSS) score | 144 | 17.07 (10.14, 24.00) | 17.31 (10.18, 24.44)^d^ |
| **Secondary Outcomes (post-intervention):** |  |  |  |
| Emotional distress (PHQ-8) score | 144 | -0.90 (-1.92, 0.12) | -1.10 (-2.11, -0.09) |
| Burnout (OLBI) score (among experienced leaders) | 108 | -0.58 (-1.83, 0.67) | -0.67 (-1.97, 0.63) |
| Volunteer satisfaction (VSI) score (among experienced leaders)^d^ | 108 | 5.16 (3.10, 7.22) | 5.01 (2.90, 7.12) |
| **Secondary Outcomes (3 months post-intervention):** |  |  |  |
| Leader self-efficacy (SGLSS) score | 145^d^ | 15.60 (9.91, 21.29)^d^ | 16.30 (10.45, 22.16) |
| Emotional distress (PHQ-8) score | 145 | -0.76 (-2.01, 0.48) | -0.96 (-2.16, 0.24) |
| Burnout (OLBI) score (among experienced leaders) | 109 | -0.85 (-1.94, 0.24) | -0.88 (-2.01, 0.24) |
| Volunteer satisfaction (VSI) score (among experienced leaders)^e^ | 108 | 3.85 (1.61, 6.10) | 3.77 (1.56, 5.98) |

CI = confidence interval; PHQ-8 = Patient Health Questionnaire-8; OLBI = Oldenburg Burnout Inventory; SMD = standardized mean difference; SGLSS = Scleroderma Support Group Leader Self-efficacy Scale; VSI = Volunteer Satisfaction Index.

^a^For the Scleroderma Support Group Leader Self-Efficacy Scale and Volunteer Satisfaction Index, positive numbers favour the intervention. For the Patient Health Questionnaire-8 and Oldenburg Burnout Inventory, negative numbers favour the intervention. ^b^Adjusted for baseline outcome score only. ^c^Adjusted for baseline score plus age (continuous; scaled), sex (male vs. female), whether the leader has scleroderma (no vs. yes), and whether the leader is a prospective vs. experienced leader. ^d^One intervention arm participant scored 118 on the Scleroderma Support Group Leader Self-Efficacy Scale (possible scores 32 to 192) at baseline, 187 post-intervention, and 32 (all items “strongly disagree”) at 3 months post-intervention. We inquired with the participant about the unusual variability in scores, and she indicated that she had intended to score all items as “strongly agree” at 3 months post-intervention (score = 192) but had mistakenly responded backwards. Thus, her score was counted as missing at 3 months post-intervention.^e^For 16 participants in intervention arm and 18 participants in waitlist control arm, item 5 was not administered due to a technical error; for those participants, total scores were calculated by multiplying total scores on 6 items administered by 7/6.
