# Supplementary material for: Effects of a support group leader education program jointly developed by health professionals and patients on peer leader self-efficacy among leaders of scleroderma support groups: a two-arm parallel partially nested randomised controlled trial
Source: Orphanet J Rare Dis. 2022 Oct 28;17:396. doi: 10.1186/s13023-022-02552-x (PMC9616616; doi:10.1186/s13023-022-02552-x)
Supplement: Supplementary file 6 — Additional file6. S6: Client Satisfaction Questionnaire-8 item and total scores. [file 13023_2022_2552_MOESM6_ESM.docx]

**Supplementary Material 6:** Post-intervention items, frequencies, and total scores for the Client Satisfaction Questionnaire-8 (CSQ-8) among participants randomised to intervention arm (N = 72; 2 participants randomised to intervention did not attend any sessions and did not complete the CSQ-8)

| **Items (1-4 points)** | **1 Point**  **N (%)** | **2 Points**  **N (%)** | **3 Points**  **N (%)** | **4 Points**  **N (%)** | **Item Mean (SD)** |
| --- | --- | --- | --- | --- | --- |
| 1. How would you rate the quality of the SPIN-SSLED Program? | 0 (0%) | 2 (2.8%) | 10 (13.9%) | 60 (83.3%) | 3.8 (0.5) |
| 2. Did the SPIN-SSLED Program provide you the kind of experience you wanted? | 0 (0%) | 1 (1.4%) | 11 (15.3%) | 60 (83.3%) | 3.8 (0.4) |
| 3. To what extent has the SPIN-SSLED Program met your needs? | 0 (0%) | 3 (4.2%) | 18 (25.0%) | 51 (70.8%) | 3.7 (0.6) |
| 4. If a friend were in need of similar help, would you recommend the SPIN-SSLED Program to him/her? | 0 (0%) | 1 (1.4%) | 5 (6.9%) | 66 (91.7%) | 3.9 (0.3) |
| 5. How satisfied are you with the amount of help you received from the SPIN-SSLED Program? | 3 (4.2%) | 2 (2.8%) | 6 (8.3%) | 61 (84.7%) | 3.7 (0.7) |
| 6. Has the SPIN-SSLED Program helped you to deal more effectively with the current COVID-19 pandemic situation? | 0 (0%) | 2 (2.8%) | 8 (11.1%) | 62 (86.1%) | 3.8 (0.4) |
| 7. In an overall, general sense, how satisfied are you with the SPIN-SSLED Program? | 1 (1.4%) | 2 (2.8%) | 7 (9.7%) | 62 (86.1%) | 3.8 (0.5) |
| 8. If you were to seek help again, would you come back to the SPIN-SSLED Program? | 2 (2.8%) | 4 (5.6%) | 6 (8.3%) | 60 (83.3%) | 3.7 (0.7) |
| **Total Score (Possible Range 8 to 32)** | Range = 16 to 32  Interquartile range = 29.25 to 32 | | |  | 30.3 (3.0) |
